# Discovery of Benzisothiazolone Derivatives as Bifunctional Inhibitors of HIV-1 Reverse Transcriptase DNA Polymerase and Ribonuclease H Activities

**DOI:** 10.3390/biom14070819

**Published:** 2024-07-09

**Authors:** Alondra Vázquez Rivera, Heather Donald, Mounia Alaoui-El-Azher, John J. Skoko, John S. Lazo, Michael A. Parniak, Paul A. Johnston, Nicolas Sluis-Cremer

**Affiliations:** 1 Department of Medicine, Division of Infectious Diseases, University of Pittsburgh School of Medicine, Pittsburgh, PA 15261, USAhld59@pitt.edu (H.D.); moa43@pitt.edu (M.A.-E.-A.); 2 Department of Chemical Biology and Pharmacology, University of Pittsburgh School of Medicine, Pittsburgh, PA 15261, USA; jskoko@gmail.com; 3 Department of Pharmacology, University of Virginia, Charlottesville, VA 22908, USA; jsl8f@virginia.edu; 4 Department of Microbiology and Molecular Genetics, University of Pittsburgh School of Medicine, Pittsburgh, PA 15219, USA; parniakm@msx.dept-med.pitt.edu; 5 Department of Pharmaceutical Sciences, University of Pittsburgh School of Pharmacy, Pittsburgh, PA 15261, USA

**Keywords:** HIV-1, reverse transcriptase, polymerase, ribonuclease H, inhibitor, benzisothiazolone

## Abstract

The ribonuclease H (RNase H) active site of HIV-1 reverse transcriptase (RT) is the only viral enzyme not targeted by approved antiretroviral drugs. Using a fluorescence-based in vitro assay, we screened 65,239 compounds at a final concentration of 10 µM to identify inhibitors of RT RNase H activity. We identified 41 compounds that exhibited 50% inhibitory concentration (i.e., IC_50_) values < 1.0 µM. Two of these compounds, 2-(4-methyl-3-(piperidin-1-ylsulfonyl)phenyl)benzo[d]isothiazol-3(2H)-one (**1**) and ethyl 2-(2-(3-oxobenzo[d]isothiazol-2(3H)-yl)thiazol-4-yl)acetate (**2**), which both share the same benzisothiazolone pharmacophore, demonstrate robust antiviral activity (50% effective concentrations of 1.68 ± 0.94 µM and 2.68 ± 0.54, respectively) in the absence of cellular toxicity. A limited structure–activity relationship analysis identified two additional benzisothiazolone analogs, 2-methylbenzo[d]isothiazol-3(2H)-one (**3**) and N,N-diethyl-3-(3-oxobenzo[d]isothiazol-2(3H)-yl)benzenesulfonamide (**4**), which also resulted in the inhibition of RT RNase H activity and virus replication. Compounds **1**, **2** and **4**, but not **3**, inhibited the DNA polymerase activity of RT (IC_50_ values~1 to 6 µM). In conclusion, benzisothiazolone derivatives represent a new class of multifunctional RT inhibitors that warrants further assessment for the treatment of HIV-1 infection.

## 1. Introduction

**Figure 1:**
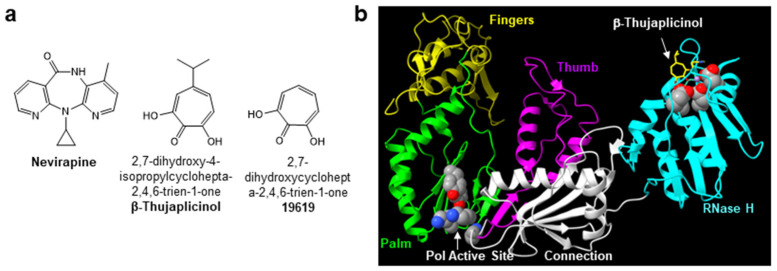
(**a**) Structures of the NNRTI nevirapine and the RNase H active site inhibitors β-thujaplicinol, a hydroxytroplone analog, and 19619. (**b**) Three dimensional structure of the p66 kDa subunit of HIV-1 RT in complex with β-thujaplicinol (pdb 3IG1).

The human immunodeficiency virus type-1 (HIV-1) encodes for the enzyme reverse transcriptase (RT) which catalyzes the conversion of the single-stranded viral RNA genome into double-stranded DNA. RT is multifunctional and exhibits both DNA polymerase and ribonuclease H (RNase H) activities [1,2]. Due to its essential role in virus replication, HIV-1 RT is a primary target for drug discovery [3,4,5]. Two therapeutic classes of RT inhibitors have been approved by the U.S. Food and Drug Administration for the prevention and treatment of HIV-1 infection. These include the nucleoside/nucleotide RT inhibitors (NRTIs, e.g., abacavir, emtricitabine, lamivudine, tenofovir disoproxil fumarate/tenofovir alafenamide and zidovudine) and the non-nucleoside RT inhibitors (NNRTIs, e.g., nevirapine (NVP; Figure 1a), efavirenz, rilpivirine, etravirine and doravirine). Within the cell, NRTIs are metabolized to their active diphosphate or triphosphate forms and compete with the natural dNTP substrate for binding and incorporation into the nascent viral DNA strand. Once incorporated, they promote the chain termination of HIV-1 DNA synthesis. NNRTIs are allosteric inhibitors of reverse transcription and bind to a hydrophobic pocket, termed the NNRTI binding pocket, that is adjacent to, but separate from, the DNA polymerase active site of RT (Figure 1b). The NNRTI binding pocket does not exist in the absence of the inhibitor, and this binding pocket is not directly involved in substrate binding or viral DNA synthesis. As a consequence, point mutations in the NNRTI binding pocket confer inhibitor resistance but do not typically impact enzyme function.

Although RT RNase H activity is critical for HIV-1 replication, the RNase H domain is the only viral enzyme not targeted by approved antiviral drugs. Several RNase H pharmacophores have been described, including diketo acid, N-hydroxyimide, hydroxytropolone (Figure 1a), pyrimidinol carboxylic acid, N-hydroxy naphthyridinone, pyrido-pyrimidinone, nitrofuran-2-carboxylic acid and thiocarbamates [6]. Most of these pharmacophores co-ordinate the metal ions at the RNase H active site (Figure 1b), and the bound and intact DNA/RNA template/primer (T/P) substrate can restrict the access of RNase H active site inhibitors, thus creating a major obstacle to RNase H inhibitor design [7]. In addition to active site inhibitors, several classes of allosteric RT RNase H inhibitors have also been identified, including acylhydrazones, 1,2,4-triazoles, thiocarbamates and vinylogous urea [6]. Of note, NNRTI binding to RT can also modulate the RNase H activity of the enzyme depending on the T/P substrate; in particular, NNRTIs accelerate the rates of polymerase-independent RNase H cleavages [8,9,10,11].

In this study, we describe the discovery of benzisothiazolone derivatives as inhibitors of both RT DNA polymerase and RNase H activity. Importantly, the active compounds demonstrate robust anti-HIV-1 activity in cell culture with minimal cytotoxicity. Consequently, benzisothiazolone derivatives are a new class of RT inhibitor that warrants further assessment for the treatment of HIV-1 infection.

## 2. Materials and Methods

### 2.1. Materials

WT (wild-type) RT, and RTs harboring the NNRTI-resistant mutations K101E, K103N, Y181C, Y188C, G190A and P236L, were overexpressed and purified to homogeneity as described previously [12]. The RT concentration was determined spectrophotometrically at 280 nm using an absorbance coefficient of 260,450 M^−1^·cm^−1^. The 7-hydroxy tropolone derivative 19,616 (2,7-dihydroxy 2,4,6-cyclo-heptatrien-1-one) was purchased from Molecular Diversity Preservation International (Basel, Switzerland). Nevirapine was purchased from Selleckchem (Houston, TX, USA). The benzisothiazolone derivatives were procured from MolPort (New York, NY, USA) or Enamine (Kyiv, Ukraine). The DNA and RNA oligonucleotides were synthesized and purified by Integrated DNA Technologies (Coralville, IA, USA). All other reagents were of the highest quality available and were used without further purification.

### 2.2. Compound Library

The NIH Molecular Library Screening Center Network (MLSCN) library of 65,239 compounds was arrayed at 10 mM concentration in DMSO into 384-well microtiter master plates and distributed to the University of Pittsburgh Molecular Library Screening Center (PMLSC) by the small molecule repository (SMR), Biofocus-DPI (A Galapagos Company, San Francisco, CA, USA). Compounds were identified by their PubChem substance identity numbers (SIDs). Daughter plates containing 2 µL of 1 mM compounds in DMSO were prepared and replicated from the MLSCN master plates using the Velocity11 Vprep (Velocity11, Menlo Park, CA, USA) outfitted with a 384-well transfer head. Aluminum adhesive plate seals were applied with an Abgene plate sealer and plates were stored at −20 °C in a Matrical MatriMinistore™ (Spokane, WA, USA) automated compound storage and retrieval system. Compound daughter plates were withdrawn from −20 °C storage, thawed at ambient temperature and centrifuged for 1–2 min at 50× *g*, and plate seals were removed prior to the transfer of 18 µL of the 50 mM Tris pH 8.0, 60 mM KCl, 5 mM MgCl_2_ assay buffer to prepare an intermediate compound library concentration of 100 µM in 1% DMSO using the Velocity11 Vprep outfitted with a 384-well transfer head. Diluted compounds were mixed by repeated aspiration and dispensation using the 384-well transfer head of the Velocity11 Vprep, and 10 µL was transferred to the compound wells of the 384-well assay plates.

### 2.3. Primary HTS Assay

Compounds were screened at a final concentration of 10 µM in 1× assay buffer (50 mM Tris pH 8.0, 60 mM KCl, 5 mM MgCl_2_) containing 1% DMSO in 384-well Greiner black polystyrene plates (VWR catalog # 82051-272). A total of 10 µL of intermediate diluted compounds (100 µM in 10% DMSO) was transferred into assay plates using the Evolution-3 (EP3) (Perkin Elmer, Waltham, MA, USA) automated liquid handling platform outfitted with a 384-well transfer head. A total of 40 µL of RT-RNase H enzyme (60 ng per well) were then dispensed into each well using the Titertek MAP-C2 reagent dispenser platform (Huntsville, AL, USA). A total of 50 µL of the RNA/DNA duplex (140 nM) substrate was then dispensed into each well using the MAP-C2 dispenser, and the assay plates were then incubated at room temperature for 20 min. After 20 min, the assay was terminated by the addition of 10 µL of 1 M EDTA pH 9.0 stop buffer. Relative fluorescent unit (RFU) signals, Excitation 490 nm and Emission 528 nm (Cutoff 515 nm), were captured on the Spectromax M5e plate reader (Molecular Devices, Menlo, CA, USA). To analyze the percent inhibition of RT-RNase H by library compounds in the primary screen, we constructed an ActivityBase™ HTS template to associate compound SIDs with the calculated percent inhibition, based on the mean maximum (1% DMSO, *n* = 32) and mean minimum (100 µM EDTA, *n* = 24) plate controls. The template also calculated the plate signal-to-background (S:B) ratios (mean maximum signal/mean minimum signal) and Z’-factor coefficient HTS performance statistics.

### 2.4. T/P Substrates

The following RNA/DNA T/P substrate was used in the present study: an 18-nucleotide RNA template (RNA-18T: 5′-GAUCUGAGCCUGGGAGCU-3′) was annealed to an 18-nucleotide primer (DNA-18T: 5′-AGCTCCCAGGCTCAGATC-3′), as described previously [13]. Fluorescein and IowaBlack FQTM were covalently attached to the 3′- and 5′-ter-mini of the RNA and DNA oligonucleotides, respectively.

### 2.5. FRET-Based RNase H Cleavage Assays

The FRET-based RNase H cleavage assays were carried out as described previously [13]. Fluorescence was measured with a SpectraMax M2 microplate spectrofluorometer (Molecular Devices, San Jose, CA, USA) using an excitation wavelength of 490 nm and an emission wavelength of 528 nm. Data were analyzed using SoftMax Pro software, version 4.7.1 (Molecular Devices, San Jose, CA, USA). The concentration of each 7-hyroxy-tropo-lone analog required to inhibit RNase H cleavage by 50% (i.e., IC_50_) was calculated using non-linear regression analyses (SigmaPlot Software Version 14, Systat Software, Inc., San Jose, CA, USA) from at least three independent experiments.

### 2.6. Fluorescence-Based DDDP Assay

DDDP assays were also performed using a QuantiFluor ONE based spectrophotometric assay to detect double-stranded DNA synthesized by recombinant HIV-1 RT [14]. The reaction contained 40 nM of a template/primer comprising a 200 nt DNA template (5′-TCTCTCTGGTTAGACCAGATCTGAGCCTGGGAGCTCTCTGGCTGACTGGGACCCA CTGCTTAA-GCCTCAATAAA-GCTTGCCTTGAGTGCTTAAAGTAGATGTGTGTGCC CGTCTGTTGTGTGACTCTGGTAACTAGAGATCCCTCAGACCCTTTTAGTCAGTGTG GAATATCTCATAGCTTGGTGCTCGAACAGTGAC-3′) annealed to an 18-nucleotide DNA primer (5′-GTCACTGTTCGAGCACCA-3′), and a mix of 50 mM Tris-HCl pH 7.8, 50 mM NaCl, 10 mM MgCl_2_ and 50 µM dNTP. The reactions were stopped after 45 min by adding the detection reagent containing 1 × Quant-Fluor One (Promega) and 12.5 mM ethylenediaminetet-raacetic acid; the samples were transferred to a 96-well black plate, and fluorescence was measured at excitation/emission (490/535 nm).

### 2.7. HIV-1 Drug Susceptibility Assays

The genes for Y181C, Y181I, Y181V and Y181F were cloned into HIV-1_LAI_. NNRTI susceptibility was determined in TZM-bl cells, as described previously [15].

## 3. Results

### 3.1. High-Throughput Screen (HTS) to Identify HIV-1 RT RNase H Inhibitors

**Figure 2:**
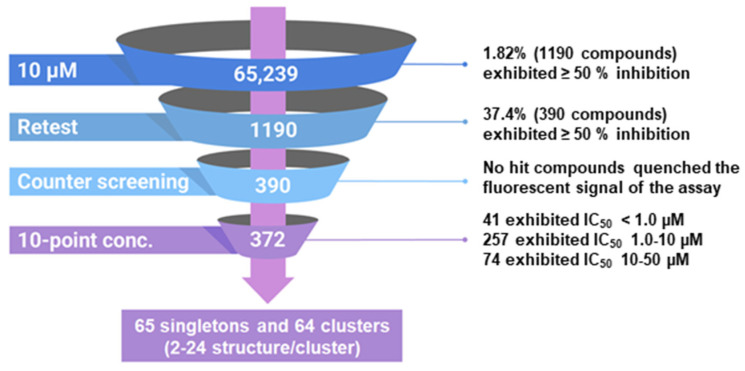
Overview of the HTS campaign to identify the RT RNase H inhibitors.

We implemented a rapid, robust and inexpensive fluorescence-based 384-well assay for the measurement of RT RNase H activity, previously described by Parniak et al. [13]. The substrate comprises an 18-nucleotide 3′-fluorescein-labeled heteropolymeric RNA annealed to a complementary 5′-dabcyl-modified DNA. RT cleaves the RNA strand of this substrate close to the 3′ end, promoting the dissociation of the fluorescein-labeled RNA fragment and generating a detectable fluorescent signal. We used this HTS assay to screen 65,239 compounds at a final concentration of 10 µM. The intra-plate Z factor ranged from 0.83 to 0.86 and the signal-to-background ratio ranged from 13.0- to 14.0-fold. The intra-plate coefficients of variation ranged between 3.7 and 5.5%, and the inter-plate coefficients of variation ranged from 4.1 to 6.1%. The screen yielded 1190 compounds with ≥50% inhibition (1.82% active rate; Figure 2). We reordered 1042 of these compounds (based on availability) that were re-tested in the primary HTS. Of these, 390 (37.4%) were confirmed as active. We then counter-screened these compounds against *fluorescein* isothiocyanate (FTIC) to confirm that they did not simply quench the fluorescence signal. None of the compounds quenched FITC fluorescence significantly (≥20%). Finally, we conducted 10-point concentration–response assays starting at 50 µM to calculate 50% inhibitory concentrations (i.e., IC_50_). Of the 390 compounds, 372 (95.4%) exhibited IC_50_ values less than 50 µM: 41 exhibited IC_50_ values < 1.0 µM; 257 exhibited IC_50_ values in the range of 1.0 to 10.0 µM; and 74 exhibited IC_50_ values in the range of 10.0 to 50.0 µM. Leadscope classification and clustering by recursive partitioning identified 65 singletons and 64 clusters ranging from 2 to 24 structures/cluster. Data from the HTS campaign were uploaded and deposited in PubChem under the indicated assay identifier (AID) numbers: primary HTS (AID 565), active confirmation and fluorescence quenching counter-screen (AID 651) and IC_50_ determinations of hits (AID 652).

### 3.2. Identification of RT RNase H Inhibitors with Antiviral Activity

**Figure 3:**
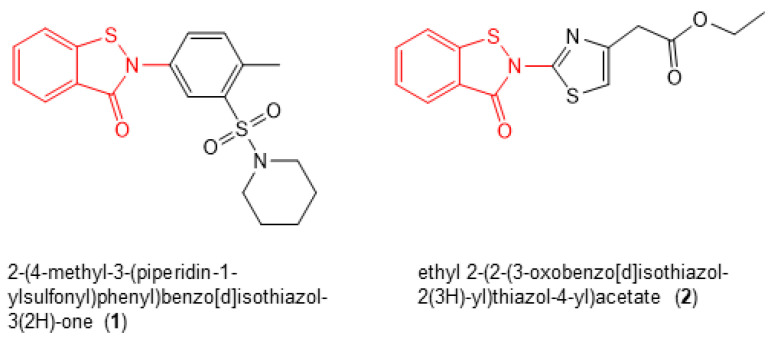
Chemical structures of compounds **1** and **2**.

Next, we re-purchased 10 hit compounds with IC_50_ values < 1.0 µM for the inhibition of RT RNase H activity that were commercially available in sufficient quantities for further analysis. We assessed their antiviral activity and cytotoxicity in TZM-bl cells, as described previously [16]. Of these compounds, only two (2-(4-methyl-3-(piperidin-1-ylsulfonyl)phenyl)benzo[d]isothiazol-3(2H)-one (**1**) and ethyl 2-(2-(3-oxobenzo[d]isothiazol-2(3H)-yl)thiazol-4-yl)acetate (**2**) (Figure 3)), which both shared the same benzisothiazolone pharmacophore (Figure 3, red), demonstrated robust antiviral activity in the absence of cellular toxicity (Table 1). The effective concentration of the compound required to inhibit 50% of HIV-1 replication in TZM-bl cells (i.e., EC_50_) and the concentration of the inhibitor that resulted in 50% TZM-bl cell death (i.e., CC_50_) were calculated to be 1.68 ± 0.94 µM and ≥100 µM for **1** and 2.68 ± 0.54 and ≥100 µM for **2**, respectively.

### 3.3. Structure–Activity Relationships (SAR)

**Figure 4:**
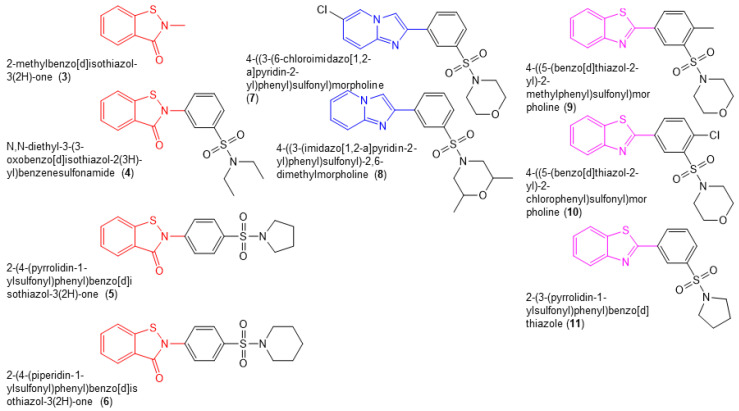
Chemical structures of compounds **3**–**11**.

We conducted a limited SAR analysis by purchasing commercially available compounds that were structurally related to **1** and **2** (Figure 4). This included four benzisothiazolone analogs (compounds **3**–**6**), two compounds in which the benzisothiazolone was replaced with an imidazo(1,2a)pyridine moiety (blue, compounds **7** and **8**) and three compounds in which the benzisothiazolone was replaced with a 1,3-benzothiazole moiety (purple, compounds **9**–**11**). We evaluated the activity of these compounds against the in vitro RNase H and DNA-dependent DNA polymerase activity of WT RT (Table 1). Of the benzisothiazolone analogs, compound **4** exhibited IC_50_ values for the inhibition of RT RNase H (IC_50_ = 0.26 ± 0.08 µM) and DNA polymerase activity (IC_50_ = 1.1 ± 0.3 µM) that were similar to **1** and **2** (Table 2). Compound **3**, which largely comprised a benzothiazolone scaffold, inhibited RT RNase H activity with an IC_50_ of 2.5 ± 0.2 µM but was completely inactive in the RT DNA polymerase assay. Compounds **3** and **4** also exhibited anti-HIV-1 activity in the absence of toxicity in TZM-bl cells (Table 1). A further two analogs, **5** and **6**, exhibited modest activity in both in vitro assays. All of the other compounds tested were inactive in both the RT RNase H and DNA polymerase assays.

### 3.4. Inhibitory Activity of 1, 2, 3 and 4 against Wild-Type (WT) HIV-1 RT and HIV-1 RT Containing NNRTI Resistance Mutations

Because NNRTI binding to RT can enhance or inhibit the RNase H activity of the enzyme depending on the T/P substrate [8,9,10,11], we assessed the capacity for **1, 2, 3** and **4** to inhibit the RNase H and DNA polymerase activity of WT RT and RT containing the NNRTI resistance mutations K101E, K103N, Y181C, Y188C, G190A and P236L (Table 2). Compound **19619** (Figure 1), a hydroxytroplone analog of *β*-thujaplicinol [17,18], was used as a positive control in the RNase H cleavage assay, and NVP (Figure 1) was used as a control in the DNA polymerase assay. Consistent with a previous study, the IC_50_ for **19619** was calculated to be 8.7 ± 3.0 µM [8]. A similar IC_50_ value was determined for all the RTs containing NNRTI-resistant mutations. NVP did not inhibit the RT RNase H cleavage activity. All four benzisothiazolone analogs inhibited the RNase H activity of WT and NNRTI-resistant RT, but small fold changes in IC_50_ (<5-fold) were noted for Y188C. Y181C RT conferred decreased susceptibility to **1** and **2**, but not **3** and **4**. K103N RT displayed 2-fold-decreased susceptibility to **1** and 3.2-fold-decreased susceptibility to **4**. In the DNA polymerase assay, compounds **1**, **2** and **4** retained activity against all RTs containing NNRTI resistance mutations. Interestingly, Y181C and Y188C RT exhibited marked increased susceptibility to **1**, **2** and **4**, a result that contrasts with the data for the inhibition of RNase H cleavage. As expected, NVP was inactive against all NNRTI-resistant RTs.

## 4. Discussion

HIV-1 RT is a primary target for antiretroviral drugs, and two distinct therapeutic classes of inhibitors, the NRTIs and NNRTIs, have been approved by the U.S. Food and Drug Administration for treatment of HIV-1 infection. Both these drug classes, however, target the DNA polymerase activity of the enzyme. Although RT RNase H activity is critical for virus replication, the RNase H domain is the only viral enzyme not targeted by approved antiviral drugs. In this study, we describe the discovery of benzisothiazolone derivatives as inhibitors of RT RNase H cleavage.

The benzisothiazolone derivatives 1 and 2 were discovered in a high-throughput screen designed to identify inhibitors of RT RNase H cleavage. Both 1 and 2 displayed potent inhibition of RT RNase H activity (IC_50_ values of 160 ± 30 nM and 130 ± 40 nM, respectively) but were also found to inhibit the enzyme’s DNA polymerase activity, albeit with IC_50_ values in the micromolar range (5.97 ± 3.10 µM and 2.64 ± 1.85 µM for 1 and 2, respectively). Typically, RNase H active site inhibitors, such as the hydroxytroplones [17,18], inhibit only the RNase H activity of RT. However, allosteric inhibitors have been reported to block both RT functions. For example, the thiocarbamates and 1,2,4-triazoles were identified in a high-throughput screen for RT RNase H inhibitors [19], but were found to also inhibit the enzyme’s DNA polymerase activity. Interestingly, computational studies and crystallography show that triazoles bind in the NNRTI binding pocket in the RT DNA polymerase domain [20]. Acylhydrazones also act as dual DNA polymerase and RNase H inhibitors. The crystal structure of dihydroxybenzoyl naphthyl hydrazone in complex with HIV-1 RT showed that the inhibitor binds to RT in the polymerase domain, near to but not within the NNRTI binding pocket [21]. Vinylogous urea compounds, also identified in a screen for RNase H inhibitors, inhibit both the DNA polymerase and RNase H activities of RT [22]. Protein footprinting and mutagenesis approaches showed that they interact with residues in the RT p51 thumb at the interface with the p66 RNase H domain [22,23]. Currently, we do not have insight into the binding site(s) or binding stoichiometry of the benzisothiazolone derivatives to RT HIV-1 RT. However, our SAR analysis revealed that compound 3 demonstrated specificity for the RNase H active site, suggesting that it may be possible to uncouple the inhibition of the DNA polymerase and RNase H activities.

There is considerable evidence that both inhibitor binding and mutations in the NNRTI-binding pocket in the RT DNA polymerase domain impact the activity of the spatially remote RT RNase H [8,9,10,11,24,25]. The mechanisms involved in this long-range alteration of RNase H activity are not entirely clear but likely involve changes in the positioning of the RNA/DNA duplex nucleic acid due to protein conformation changes in the polymerase domain following NNRTI binding [8]. The effect of NNRTIs on RT RNase H activity, however, is typically much less than on RT DNA polymerase activity. In this study, we assessed the activity of the benzisothiazolone derivatives against RTs containing NNRTI resistance mutations. The hydroxytroplone 19619, which binds at the RNase H active site of RT, retained activity against all NNRTI-resistant RTs. In general, the benzisothiazolone derivatives inhibited the RNase H activity of all RTs tested, but we did observe that some mutations, such as Y188C, decreased inhibitor potency. Of note, the activity of the benzisothiazolone derivatives against the DNA polymerase activity of the NNRTI-resistant RTs was not reduced by any of the mutations tested. Instead, we report that Y188C RT was hyper-susceptible to all of the analogs tested in the polymerase assay. While these data highlight that mutations in the NNRTI-binding pocket modulate activity of the benzisothiazolone analogs, they do not provide definitive proof that they bind to this pocket. In addition to the NNRTI-binding pocket, other allosteric binding sites in RT have been identified, including the NNRTI-Adjacent, Knuckles, Incoming Nucleotide Binding, 399, 428, 507 and RNase H Primer Grip-Adjacent binding sites [26]. Of these sites, compounds that bind to the Knuckles, Incoming Nucleotide Binding, 507 and NNRTI-Adjacent sites were reported to be inhibitory toward both the DNA polymerization and RNase H activities [26]. Furthermore, Corona et al. identified an isatin-based compound (RMNC6) that acted as a bifunctional inhibitor of RT DNA polymerase and RNase H activity, and molecular docking studies suggested two binding sites in RT: one at a site partially overlapping the NNRTI-binding site; and a second at a site close to the RNase H active site [27]. Sonar et al. reported that N-oleycaffeamide was a dual inhibitor of HIV-1 RT and molecular modeling studies indicated similar binding sites to those described for RMNC6 [28]. Several other compounds were also reported to inhibit both the DNA polymerase and RNase H activities of RT by binding to allosteric sites in the enzyme, including 5-nitro-3-(2-(4-phenylthiazol-2-yl)hydrazineylidene)indolin-2-one derivatives [29]. We hypothesize that the benzisothiazolone analogs described in this study similarly bind to allosteric sites in RT, and we are currently trying to identify these binding site(s) using structural biology approaches.

In conclusion, we identified benzisothiazolone derivatives as inhibitors of the RNase H activity of HIV-1 RT, but they also block the enzyme’s DNA polymerase activity. Importantly, these benzisothiazolone derivatives demonstrate robust anti-HIV-1 activity in cell culture with minimal cytotoxicity. Consequently, benzisothiazolone derivatives are a new class of RT inhibitor that warrants further assessment for the prevention and/or treatment of HIV-1 infection.

## Figures and Tables

**Table 1:** Activity of **1**–**11** against purified RT and HIV-1. The data represent the mean ± standard deviation from at least three independent experiments.

	RNase H Activity	Polymerase Activity	Antiviral Activity	Cytotoxicity
	IC_50_ (µM)	IC_50_ (µM)	EC_50_ (µM)	CC_50_ (µM)
	RNase H	DNA Polymerase	HIV-1	
**1**	0.16 ± 0.03	5.97 ± 3.10	1.68 ± 0.94	≥100
**2**	0.13 ± 0.04	2.64 ± 1.85	2.68 ± 0.54	≥100
**3**	2.5 ± 0.2	>81	28.45 ± 12.26	≥100
**4**	0.26 ± 0.08	1.1 ± 0.3	3.27 ± 0.49	≥100
**5**	15.41 ± 9.2	25 ± 25		
**6**	39.21 ± 20.99	12.2 ± 3.7		
**7**	>81	>81		
**8**	>81	>81		
**9**	>81	>81		
**10**	>81	>81		
**11**	>81	62 ± 38		

**Table 2:** Inhibition of WT and NNRTI-resistant HIV-1 RT RNase H and DNA-dependent DNA polymerase activity by **1** and **2**. The data represent the mean ± standard deviation from at least three independent experiments.

Inhibition of RT RNase H Activity										
RT	19619		1		2		3		4	
	IC_50_ (µM)	Fold-R ^1^	IC_50_ (µM)	Fold-R	IC_50_ (µM)	Fold-R	IC_50_ (µM)	Fold-R	IC_50_ (µM)	Fold-R
WT	8.7 ± 3.0	-	0.16 ± 0.03	-	0.13 ± 0.04	-	2.5 ± 0.2		0.26 ± 0.08	
Y181C	12.1 ± 1.6	1.4	0.47 ± 0.05	2.9	0.55 ± 0.02	4.2	2.1 ± 0.3	0.8	0.27 ± 0.03	1.0
K103N	9.4 ± 0.9	1.1	0.34 ± 0.04	2.1	0.24 ± 0.12	1.8	2.4 ± 0.2	1.0	0.83 ± 0.23	3.2
G190A	7.1 ± 1.9	0.8	0.21 ± 0.02	1.3	0.23 ± 0.02	1.8	2.4 ± 0.4	1.0	0.31 ± 0.04	1.2
K101E	8.1 ± 1.7	0.9	0.25 ± 0.06	1.6	0.28 ± 0.02	2.2	2.2 ± 0.3	0.9	0.09 ± 0.03	0.3
Y188C	5.5 ± 2.4	0.6	0.32 ± 0.01	2.0	0.59 ± 0.04	4.5	5.7 ± 1.2	2.3	1.10 ± 0.32	4.2
P236L	8.9 ± 2.5	1.0	0.22 ± 0.01	1.4	0.21 ± 0.01	1.6	2.2 ± 0.2	0.9	0.31 ± 0.04	1.2
**Inhibition of RT DNA polymerase activity**										
	**NVP**		**1**		**2**		**3**		**4**	
WT	4.96 ± 3.19	-	5.97 ± 3.10	-	2.64 ± 1.85	-	-		1.1 ± 0.3	-
Y181C	>81	>16	0.61 ± 0.06	0.1	0.71 ± 0.10	0.3	-		0.5 ± 0.2	0.45
K103N	>81	>16	3.66 ± 1.84	0.6	3.28 ± 1.53	1.2	-		2.1 ± 0.7	1.9
G190A	>81	>16	1.60 ± 0.88	0.2	1.93 ± 0.49	0.7	-		1.7 ± 1.0	1.5
K101E	>81	>16	7.33 ± 7.05	1.2	4.88 ± 1.56	1.8	-		1.8 ± 0.7	1.6
Y188C	>81	>16	0.14 ± 0.01	0.02	0.53 ± 0.08	0.2	-		0.3 ± 0.1	0.3
P236L	>81	>16	2.22 ± 0.18	0.4	5.13 ± 1.68	1.9	-		1.6 ± 0.9	1.5

^1^ Fold-R; fold resistance; IC_50_^(mutant)^/IC_50_^(WT)^.

## Data Availability

All raw data are available from the authors upon request.
